# Host-induced silencing of *Fusarium culmorum* genes protects wheat from infection

**DOI:** 10.1093/jxb/erw263

**Published:** 2016-08-18

**Authors:** Wanxin Chen, Christine Kastner, Daniela Nowara, Ely Oliveira-Garcia, Twan Rutten, Yusheng Zhao, Holger B. Deising, Jochen Kumlehn, Patrick Schweizer

**Affiliations:** ^1^Leibniz-Institut für Pflanzengenetik und Kulturpflanzenforschung (IPK) Gatersleben, Corrensstrasse 3, D-06466 Stadt Seeland, Germany; ^2^Martin-Luther Universität Halle-Wittenberg, Phytopathologie und Pflanzenschutz, Betty Heimann Straße 3, D-06120 Halle, Germany

**Keywords:** *Barley stripe mosaic virus*, chitin synthase, β-1,3-glucan synthase, HIGS, lipase, MAP kinase.

## Abstract

Plants producing antisense or double-stranded RNA molecules that target specific genes of eukaryotic pests or pathogens can become protected from their attack. This beneficial effect was also reported for plant–fungus interactions and is believed to reflect uptake of the RNAs by the fungus via an as yet unknown mechanism, followed by target gene silencing. Here we report that wheat plants pre-infected with *Barley stripe mosaic virus* (BSMV) strains containing antisense sequences against target genes of the *Fusarium* head blight (FHB) fungus *F. culmorum* caused a reduction of corresponding transcript levels in the pathogen and reduced disease symptoms. Stable transgenic wheat plants carrying an RNAi hairpin construct against the β-1, 3-glucan synthase gene Fc*Gls1* of *F. culmorum* or a triple combination of Fc*Gls1* with two additional, pre-tested target genes also showed enhanced FHB resistance in leaf and spike inoculation assays under greenhouse and near-field conditions, respectively. Microscopic evaluation of *F. culmorum* development in plants transiently or stably expressing Fc*Gls1* silencing constructs revealed aberrant, swollen fungal hyphae, indicating severe hyphal cell wall defects. The results lead us to propose host-induced gene silencing (HIGS) as a plant protection approach that may also be applicable to highly FHB-susceptible wheat genotypes.

## Highlight

Wheat plants transiently or stably accumulating antisense or double-stranded RNA, which are directed against essential genes of the *Fusarium* head blight fungus *F*. *culmorum*, were more resistant against the disease.

## Introduction

Many eukaryotic organisms including higher plants possess miRNA- or siRNA-expressing genes that contribute to the regulation of developmental or stress-related processes via transcriptional or post-transcriptional gene silencing (Brodersen and Voinnet, 2006; Frizzi and Huang, 2010). One of the most important and well-known functions of siRNA-mediated RNAi is the targeting and degradation of viral RNA, which has been counteracted by the evolution of viral silencing suppressors (Pumplin and Voinnet, 2013). In recent years it has become evident that siRNAs can also be transferred between plants and eukaryotic pathogens or pests, which may result in the targeting of transcripts of the interacting organism and, finally, gene silencing. Gene silencing in pathogens or pests by interfering RNAs derived from engineered plant genomes is referred to as host-induced gene silencing (HIGS) or host-mediated RNAi, and was reported to act on nematodes, chewing insects, parasitic plants, fungi, and oomycetes (Huang *et al.*, 2006; Mao *et al.*, 2007; Roney *et al.*, 2007; Nowara *et al.*, 2010; Tinoco *et al.*, 2010; Yin *et al.*, 2011; Nunes and Dean, 2012; Zhang *et al.*, 2012; Koch *et al.*, 2013; Panwar *et al.*, 2013; Pliego *et al.*, 2013; Ghag *et al.*, 2014; Baulcombe, 2015; Cheng *et al.*, 2015; Govindarajulu *et al.*, 2015). Therefore, HIGS offers a new, pesticide-free and potentially sustainable option of plant protection, provided sufficiently efficient down-regulation of transcript abundance can be reached.

*Fusarium* head blight (FHB) caused mostly by the two fungal species *F. graminearum* and *F. culmorum* is a serious disease in wheat, barley, and maize resulting in an annual loss of wheat yield equivalent to more than US$ 1 billion just in the USA and the Great Northern Planes in years with high disease pressure (Chakraborty and Newton, 2011; Scherm *et al.*, 2013). Fungi causing FHB also produce a number of toxins belonging to the class of trichothecenes, which are hazardous to humans and animals and can cause the degradation of harvest lots of premium wheat to biofuel material (Cheli *et al.*, 2014). The problem of FHB is aggravated by the fact that current fungicides possess limited efficacy against the disease and that no major resistance (R)-genes have been identified so far in the most heavily affected cereals wheat and barley. Instead, plant resistance appears to be based on a rather large number of quantitative trait loci (QTLs) with small to intermediate effects (Liu *et al.*, 2009; Loffler *et al.*, 2009). The complex genetic set-up of FHB resistance represents a considerable challenge to breeders and slows down progress due to linkage drag when using exotic resistance donors such as old landraces that carry many undesired traits. Therefore, biotechnological solutions to durable FHB resistance might be an attractive alternative. So far, transgenic approaches in wheat for FHB resistance mainly focused on the constitutive overexpression of plant genes encoding candidate proteins for defense signaling or cell wall-related defense responses (Makandar *et al.*, 2006; Shin *et al.*, 2008; Volpi *et al.*, 2011; Xiang *et al.*, 2011; Ferrari *et al.*, 2012; Zhu *et al.*, 2012). Such overexpression approaches, despite having resulted in partial protection, bear the risk of being energy demanding or having unwanted side effects on growth of non-infected plants (Gurr and Rushton, 2005; Goto *et al.*, 2016). HIGS offers an attractive alternative to overexpression of defense-related genes for achieving durable plant protection because, by definition, it is expected not to interfere with plant genomes or derived products due to the sequence specificity of the RNAi constructs directed against a pest or a pathogen. Therefore, undesired side effects of HIGS-mediating transgenes are expected to be rare.

Here we describe an application of HIGS against *F. culmorum* in plants previously infected with the transient silencing vector *Barley stripe mosaic virus* (BSMV) carrying target gene fragments of *F. culmorum* in antisense orientation (Holzberg *et al.*, 2002). We also tested the effect of stably integrated RNAi hairpin constructs in transgenic wheat. Both transient and stable silencing of three out of four selected fungal targets resulted in reduced fungal transcript levels, disease symptoms, and fungal biomass. The results are discussed with respect to prospects and limitations of HIGS to control the FHB disease.

## Materials and methods

### Plants and fungus

Spring wheat (cv. Apogee) was grown in 11×11×12cm pots in a climatized greenhouse at 22 °C and 16h light, with one dose of pellet fertilizer (Osmocote; Scotts Co., Australia) given at the onset of shoot elongation (Zadok’s stage 30–31). Fungicide and insecticide treatments were given at the time of fertilization. The single-spore-derived, highly virulent Dutch isolate FC46 (IPO 39-01) of *F. culmorum* (Miedaner *et al.*, 2014) was maintained as a stock by keeping a piece of mycelium-overgrown potato dextrose ager (PDA) in sterilized water at 4 °C. For plant inoculations, 100 µl of the stock was spread onto PDA and incubated in a growth chamber with 16h daylight at 20 °C for 7–10 d, then a piece of fresh mycelium was transferred onto synthetic nutrient-poor SNA agar (Nirenberg, 1981) for 2–4 weeks under black light to produce large amounts of macroconidia.

### DNA constructs

BSMV is a positive-sense RNA virus, containing α, β, and γ fragments. The constructs of BSMV cDNA clones utilized in this study were described in Bruun-Rasmussen *et al.* (2007), with the recombinant γ-genome carrying antisense sequences of target genes. Using gene-specific primers FGL1-F1 and FGL1-R1 derived from the *F. graminearum* gene FGSG_05906.3 in the Fusarium Graminearum Genome DataBase (http://pedant.helmholtz-muenchen.de/pedant3htmlview/pedant3view?Method=analysis&Db=p3_p13839_Fus_grami_v32), a 606bp sequence of the *F. culmorum Fgl1* gene was amplified and cloned into pBluescript II SK, then, by double digestion by *Eco*RV and *Pst*I, a 461bp *Fgl1* gene fragment was inserted into the T7-γ vector in antisense orientation to generate T7-γ-Fgl1 (Table 1; Supplementary Table S1 at *JXB* online). Fragments used to silence Fc*Fmk*1 (primers derived from FGSG_06385 of *F. graminearum*), Fc*Gls*1 (primers derived from FGSG_07946.3 of *F. graminearum*) and Fc*Chs*V (primers derived from FGSG_01964.3 of *F. graminearum*) were obtained in a similar way by PCR amplification with corresponding primer pairs from *F. culmorum* cDNA and blunt insertion into *Sma*I-digested T7-γ and by selecting the antisense orientation to obtain T7-γ-Fmk1, T7-γ-GS1, and T7-γ-ChsV vectors.

**Table 1:** Summary of HIGS target genes in *F. culmorum*

Gene	Encoded protein	*F. graminearum* gene ID^*a*^	FL^*b*^ including introns (bp)	*F. culmorum* accession numbers	BSMV-HIGS fragment (bp)	Target site in FL sequence
Fc*Fgl1*	Secreted lipase	FGSG_05906.3	1268	KP195142	461	607–1067
Fc*Fmk1*	Mitogen-activated protein (MAP) kinase	FGSG_06385.3	1712	KP195139	300	633–932
Fc*Gls1*	β-1,3-Glucan synthase	FGSG_07946.3	6792	KP195140	300	3594–3893
Fc*ChsV*	Chitin synthase V, myosin-motor domain	FGSG_01964.3	5703	KP195141	385	3146–3530

^*a*^ In the ‘Fusarium Comparative Database’ (http://www.broadinstitute.org/annotation/genome/fusarium_group/MultiHome.html).

^*b*^ FL, full-length sequence from the translation start to stop codon of the corresponding *F. graminearum* genomic sequence.

A 500bp fragment of Fc*Gls1* was PCR amplified from *F. culmorum* cDNA and inserted as a blunt-ended fragment into the *Swa*I site of the Gateway entry vector pTA38 to generate pTA38-FcGls1 (500). The *Gls1* fragment was recombined into the generic binary vector pIPKb027 by the Gateway LR reaction to obtain pIPKb027-FcGls1.

For pTA38-FcGls1-FcFmk1-FcChsV, the 300bp PCR fragment of Fc*Gls1*was inserted into the *Swa*I site of pTA38, followed by serial digestion and ligation (*Eco*RV and *Hin*dIII, respectively) to insert also the 299bp PCR fragment of Fc*Fmk1* and the 385bp PCR fragment of Fc*Chs*V, respectively. The Gls1-Fmk1-ChsV fragment was then recombined by the Gateway LR reaction into pIPKb027 (accession no. KJ508866) to generate pIPKb027-FcGls1-FcChsV-FcFmk1.

For the RNAi reporter assay, a *Swa*I site was introduced into pIPKTA26 (see Supplementary Fig. S1; Dong *et al.*, 2006) between the ORF of a destabilized ACC synthase:GFP (green fluorescent protein) protein and the transcription terminator to allow easy transcriptional fusion of target sequences. A 500bp Fc*Gls1* fragment, with sequence identical to that in the binary vector pIPK027-FcGls1, was introduced into the *Swa*I site, resulting in construct pIPKTA26_Gls1.

### RNA isolation, RT–PCR, and real-time PCR

Plant tissue was frozen and ground to powder in liquid nitrogen, and total RNA was isolated using Trizol reagent (Invitrogen). Following DNase I treatment to remove genomic DNA contamination, first-strand cDNAs were synthesized using the oligo(dT)_18_ primer and RevertAid Reverse Transcriptase (Thermo-Scientific) according to the manufacturer’s protocols. Reverse transcription–PCR (RT–PCR) was performed in a 20 µl volume of PCR Master Mix (Thermo-Scientific) using a Biometra PCR machine (Whatman). A 10 µl final volume of SYBR Green or Taqman master mix (Thermo-Scientific) was used for quantitative real-time PCR in an ABI 7900HT fast real-time PCR system (Applied Biosystems/Life Technologies). After 40 thermal cycles, transcript levels were quantified by using SDS 2.2.1 software (Applied Biosystems/Life Technologies) according to the standard curve method. The relative quantity of HIGS target transcripts was calculated by normalization of absolute quantities to the internal control gene encoding translation elongation factor 1 (Fc*EF1*) of *F. culmorum* (accession no. JF740860.1).

### Generation of transgenic wheat plants

The binary vectors were transferred to *Agrobacterium tumefaciens* strain AGL-1 used to inoculate immature wheat embryos. The generation of transgenic plants and their molecular analysis for transgene integration was conducted as previously described by Hensel *et al*. (2009).

### Spike inoculation with *F. culmorum*

For point inoculation of BSMV-HIGS plants in the greenhouse or in the near-field trial, spores were freshly washed with H_2_O (0.05% Tween-20) and diluted to 50 spores µl^–1^, followed by pipetting of 10 µl of the suspension into one wheat floret at the stage of anther extrusion. Two flowering spikelets in the middle of each spike were inoculated, labeled by a black dot, and covered by a moist, transparent plastic bag for 48h. The numbers of blighted as well as total spikelets was recorded from 7 d to 21 d after inoculation. Disease spreading as a percentage was calculated as 100×(blighted spikelets–2)/(total spiklets–2).

### Detached leaf inoculation with *F. culmorum*

The detached leaf assay with *F. culmorum* was performed using a modification of the method of Kumar *et al.* (2011). Fourteen- or 18-day-old fully extended second or third leaves of seedlings were used for the detached leaf assay. Leaves were cut into 2.5cm long segments and put into microtiter-format plates (Greiner Bio-One: order no. 96077307) half-filled with 1% (w/v) Phytoagar (Duchefa: P1003.1000) containing 0.002% (w/v) benzimidazole (Fluka: 12250) with the adaxial side facing up. After prick wounding with a thin needle, leaf sections were point-inoculated with 10 µl droplets of a 5×10^5^ conidia ml^–1^ suspension of *F. culmorum*. Plate covers were sprayed with water and sealed properly to maintain high humidity, and cultivated in a growth chamber with 16h daylight at 22 ° C for 4–5 d. FHB symptoms (brown lesions) were photographed, and lesion size was measured using ImageJ software (Abramoff *et al.*, 2004). Ten to 16 detached leaf sections of each genotype were analyzed in three independent biological replicates (inoculation experiments).

### RNAi reporter assay by unstable GFP

Microprojectile bombardment of wheat and barley leaf segments and the assay of unstable GFP expression were performed as described (Dong *et al.*, 2006), with modifications. Plasmids pIPKTA26 (empty vector control) or pIPKTA26_Gls1 were co-bombarded with pUbiGUS (for normalization of transformation efficiency; Schweizer *et al.*, 1999) into split segments of second leaves of 14-day-old seedlings. For the measurement of silencing activity of HIGS transgenic plants, cells expressing destabilized GFP were counted 24–48h after the bombardment. β-Glucuronidase (GUS) staining was then performed and the GUS-stained cell numbers were counted as an internal control of transformation efficiency. For proof of concept, a transient silencing experiment was performed in barley by co-bombarding the RNAi reporter pIPKTA26_Mlo with the RNAi construct piPKTA30N_Mlo, except that the number of GFP-fluorescing cells was normalized to the number of anthocyanin-accumulating cells by co-bombarding pBC17 as a third component, (Dong *et al.* 2006).

### BSMV-mediated VIGS and HIGS

The BSMV-based virus-induced gene silencing (VIGS) method was modified from a previous report (Bruun-Rasmussen *et al.*, 2007). pBluescript-based T7 plasmids containing cDNAs of BSMV subgenomic components were linearized by *Mlu*I digestion (T7-α and T7-γ recombinant) or *Spe* I (T7-β) and used as templates for *in vitro* transcription following the manual of the mMessage mMachine kit (Ambion). The three capped viral RNAs were mixed in equal amounts (~60 µg of each) and rubbed into the flag leaf together with 40 µl of FES buffer [77mM glycine, 60mM K_2_HPO_4_, 22mM Na_4_H_2_P_2_O_7_, 1% (w/v) bentonite, and 1% (w/v) Celite, pH 8.9].

### Greenhouse trials of transgenic plants

Transgenic wheat plants were grown in pots of 16cm diameter containing soil of the IPK nursery in a standard greenhouse at ~22 °C with 16h daylight supplemented by artificial light, with a single fertilizer (Osmocote; Scotts Co.) treatment 2 weeks after sowing, and a single fungicide (Vegas; BASF, Limburgerhof, Germany) and insecticide (Perfekthion; BASF) treatment when the corresponding infection appeared.

The near-field trial was conducted in three semi-open greenhouses, each containing two plots inside. Transgenic, azygous, and wild-type (cv. Bobwhite) 3-week-old wheat seedlings were planted in a fully randomized design per plot and surrounded by guiding rows of wild-type Bobwhite.

### Statistical data evaluation

For both BSMV-HIGS and the standard greenhouse trials, spreading disease severity and relative quantity of HIGS target transcripts per individual plant were normalized to corresponding mean values of BSMV:00-inoculated, wild-type, or azygous control plants, followed by the addition of a constant factor of 0.01 (to avoid zero values of completely resistant or silenced individuals) and log(2) transformation. A two-tailed one-sample *t*-test or Wilcoxon signed rank was applied against corresponding hypothetical control values, depending on normal/non-normal data distribution.

For the near-field trial in semi-open greenhouses, disease severity was calculated as a percentage of blighted spikelets, normalized to the mean value of pooled azygous control lines and log(e) transformed. Transgene effects were assessed by two-tailed, unpaired *t*-test and by one-way ANOVA. Means and SDs of disease severity of transgenic, azygous, and wild-type plants for each day after inoculation are described in Supplementary Table S2. The ANOVA and Mann–Whitney’s test were done for the raw data under the assumption of normal and non-normal data distribution, respectively. For the log(e)-transformed data, the Wilcoxon signed rank test as well as the ANOVA are used for the same reason. The ANOVA followed by a Tukey’s HSD (honest significant difference) test is applied to the log(e)-transformed data in order to show the significant difference for pairwise comparison between transgenic, azygous, and wild-type plants. Multiple analysis of variance (MANOVA) was also applied to the raw data to investigate the influence of different effects in the experimental design.

### Wheat germ agglutinin staining and confocal laser scanning microscopy

After *Fusarium* inoculation, wheat spikelets were destained in fixative solution [absolute ethanol:chloroform (1:4), 0.15% trichloracetic acid)] for at least 48h and stored until use. For longer term storage, samples were kept in 50% glycerol at 4 °C. For wheat germ agglutinin staining, tissues were first washed and incubated in phosphate-buffered saline (PBS; 137mM NaCl, 2.7mM KCl, 10mM Na_2_HPO_4_·2H_2_O, 2mM KH_2_PO_4_, pH 7.4) for 20min, then transferred into staining solution [PBS containing 1% wheat germ agglutinin–Alexa Fluor^®^ 488 (WGA–AF 488) at 1 µg µl^–1^ (Life Technologies, Germany), and 1% BSA (1 µg µl^–1^, Sigma)] and vacuum infiltrated for 20min. Samples were further incubated for at least 48h at 4 °C in the dark until microscopy. Confocal fluorescence images from BSMV-treated plants were recorded on a Nikon Eclipse 90i confocal laser scanning microscope (Nikon, Düsseldorf, Germany) with the following settings: laser light transmittance, 25% (ND4 in, ND8 out); pinhole diameter, 30mm; lens, Plan Apo 60/1.4 oil lens. WGA–AF 488 fluorescence was excited with a 488nm laser line and detected at 505–540nm. Samples from transgenic plants were analyzed on a Zeiss LSM 510 META confocal laser scanning microscope. Recordings were made with a ×40 objective (NA 1,2). WGA–AF 488 fluorescence was recorded with a 488nm laser line in combination with a 505–550nm bandpass filter.

## Results

### BSMV-mediated VIGS in wheat spikes

**Fig. 1.**
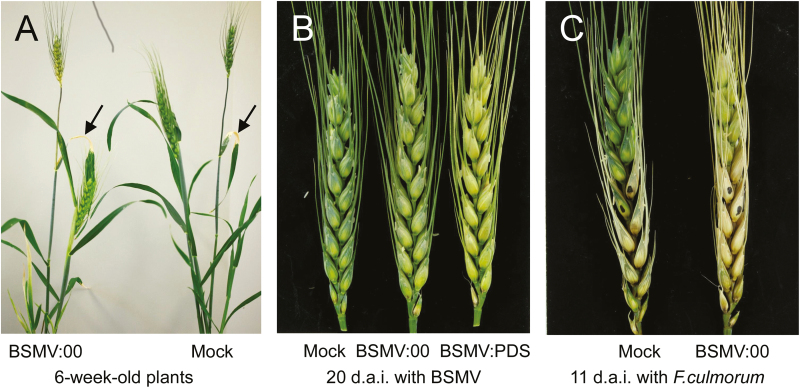
Virus-induced gene silencing in spikes of adult wheat plants mediated by the *Barley stripe mosaic virus* (BSMV). (A) Adult wheat plants 14 d after BSMV or mock inoculation on the flag leaf (arrows). (B) Moderate photobleaching in glumes induced by a BSMV antisense construct targeting phytoene desaturase (PDS) gene(s) of wheat. (C) Successful, spreading infection by *F. culmorum* of BSMV-pre-inoculated wheat plants. (A–C) BSMV:00, empty vector containing the multiple cloning site without the antisense DNA fragment.

**Fig. 2.**
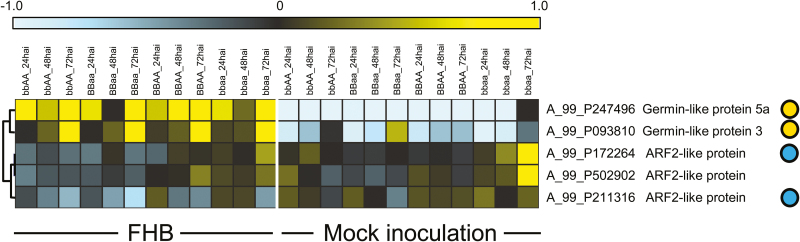
Transcriptional down-regulation of *Auxin response factor 2* (*ARF2*)-like genes in wheat after inoculation with *F. culmorum*. Hierarchical clustering of transcript levels [quantile normalized and log(2) transformed] derived from the 44K Wheat Gene Expression microarray of Agilent. Yellow and blue dots highlight probes detecting significantly up- or down-regulated transcripts, *P*<0.05 in a paired *t*-test between all inoculated and mock-treated samples. Probe A_99_P502902 corresponds to TC432968 in the TIGR wheat gene index; probes A_99_P172264 and A_99_P211316 correspond to TC412880 and ‘*TaArf2*’ (accession no. AY902381). The color code spans the log(2)-transformed, median-centered signal intensities per gene from –1 to 1. RNA sample labeling: bbAA_NNhai, pooled BC_3_ introgression lines of wheat cv. Opus containing the *Fhb5* resistance QTL from landrace Sumai 3 on chromosome 5A, sampled at NN hours after inoculation; BBaa_time, pooled BC_3_ introgression lines of wheat cv. Opus containing the *Fhb1* resistance QTL from landrace Sumai 3 on chromosome 3BS; BBAA_time, pooled BC_3_ introgression lines of wheat cv. Opus carrying both QTLs; bbaa_time, introgression lines with both QTLs crossed out. For a detailed description of the introgression lines, see von der Ohe *et al.* (2010).

**Fig. 3.**
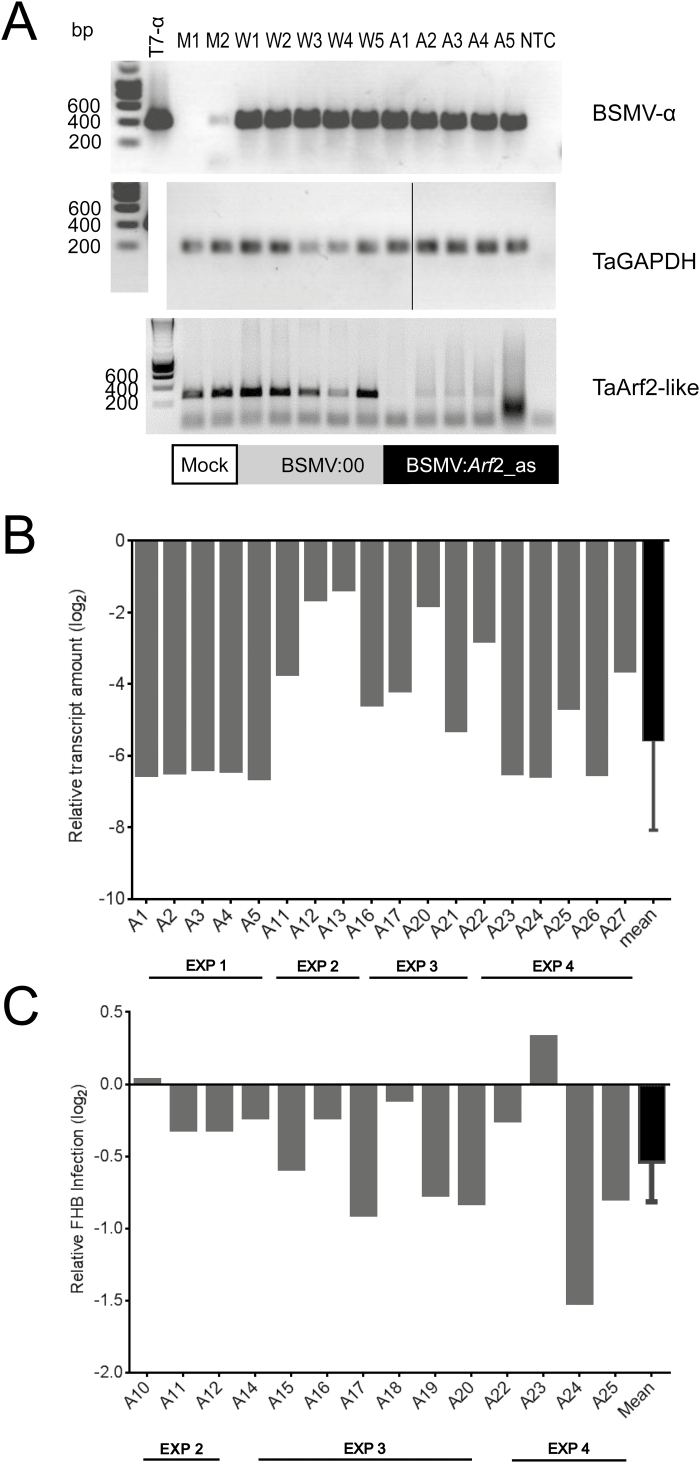
VIGS of *Auxin response factor 2*-like genes reduces susceptibility of spikes to *F. culmorum* attack. (A) BSMV infection and strong target gene silencing of wheat spikes revealed by real-time quantitative PCR (RT-qPCR) using primers for a partial α-genome fragment of the virus, a Ta*Arf* 2-like fragment outside the target sequence of the VIGS construct, and glyceraldehyde-3-phosphate dehydrogenase (TaGAPDH) as non-targeted control. The black vertical line indicates the borders of two assembled, separated parts of the same gel photographed subsequently under identical illumination and exposure conditions. For the entire, unassembled gel image, see also Supplementary Fig. S8. (B) RT-qPCR of Ta*Arf2-like* mRNA levels [relative to TaGAPDH and log(2) transformed] in VIGS spikes from four independent experiments showed significant knock-down compared with BSMV:00 controls (*P*<0.0001, one-sample *t*-test). (C) FHB infection of BSMV:Arf 2_as-treated spikes [relative to BSMV:00 controls and log(2) transformed] showed significant reduction over three independent inoculation experiments (*P*=0.0026). Abbreviations: M, mock-inoculated; W, BSMV:00-inoculated; A, BSMV:Arf2_as-inoculated.

Rubbing a 1:1:1 mixture of BSMV α, β, and γ *in vitro* transcripts into flag leaves of the early flowering, extreme dwarf wheat cv. Apogee caused the expected, although rather mild, photobleaching phenotype in the lemma and palea of spikes, if the γ-RNA contained an antisense fragment of the wheat *phytoene desaturase* (Ta*Pds*) gene suggesting systemic spread of the virus into vegetative spike tissues (Fig. 1B). No disease symptoms were observed in spikes of plants treated with the control virus strain BSMV:00, which carries the empty multiple-cloning site behind the ORF of the γ-b gene (Supplementary Fig. S2). As shown in Fig. 1C, pre-inoculation with BSMV:00 did not prevent successful infection by *F. culmorum*. In contrast, we observed a trend for enhanced FHB disease symptoms in BSMV:00-pre-inoculated plants compared with mock-treated plants. An influence of BSMV pre-infection on plant–fungus interactions has been described before (Tufan *et al.*, 2011). It was therefore important always to compare VIGS effects with the BSMV:00- and not the mock-treated controls. In order to provide additional proof of concept for BSMV-mediated transient gene silencing in spikes, we assayed transcript levels of the wheat *Arf2* gene (GenBank accession no. AY902381) encoding an auxin response factor 2-like protein, which represents the closest homolog of At*ARF2* in *Arabidopsis thaliana* (Okushima *et al.*, 2005). Wheat *Arf2-like* was chosen as the silencing target because it is well expressed in wheat spikes and because the closest homolog in *A. thaliana* plays important roles not only in auxin signaling but also in signal crosstalk with the gibberellin and brassinosteroid pathways with their proposed central function during plant–pathogen interactions (Vert *et al.*, 2008; Albrecht *et al.*, 2012; Belkhadir *et al.*, 2012; Saville *et al.*, 2012; Richter *et al.*, 2013). Therefore, by altering *Arf2-like* expression, we might also influence the interaction of FHB fungi with wheat. The idea was supported by the observation that two predicted *ARF2-like* VIGS targets were—moderately but significantly—transcriptionally down-regulated in *F. culmorum*-inoculated wheat spikes (Fig. 2; Supplementary Table S3). For comparison, two significantly up-regulated wheat transcripts encoding germin-like proteins were also included in the hierarchical clustering. Both *Arf2-like* transcripts are VIGS targets according to *in silico* prediction by the si-Fi software (http://labtools.ipk-gatersleben.de/index.html; Supplementary Fig. S3). As shown in Fig. 3A and B, *TaArf2-like* mRNA levels were strongly reduced by BSMV:Arf2_as. The presence of BSMV α-RNA in all analyzed spikes of virus-infected plants demonstrates efficient systemic virus spread after flag leaf inoculation. The silencing of *Arf2-like* genes was associated with a moderate but significant reduction in FHB disease symptoms (Fig. 3C), supporting the idea that *F. culmorum* might benefit from auxin signaling for host colonization, as described for other pathogens (Fu *et al.*, 2011; Cui *et al.*, 2013). In conclusion, BSMV-mediated transient gene silencing in spikes of adult wheat plants appears suitable to assess candidate genes for resistance or susceptibility to FHB.

### BSMV-mediated HIGS in *F. culmorum*

**Fig. 4.**
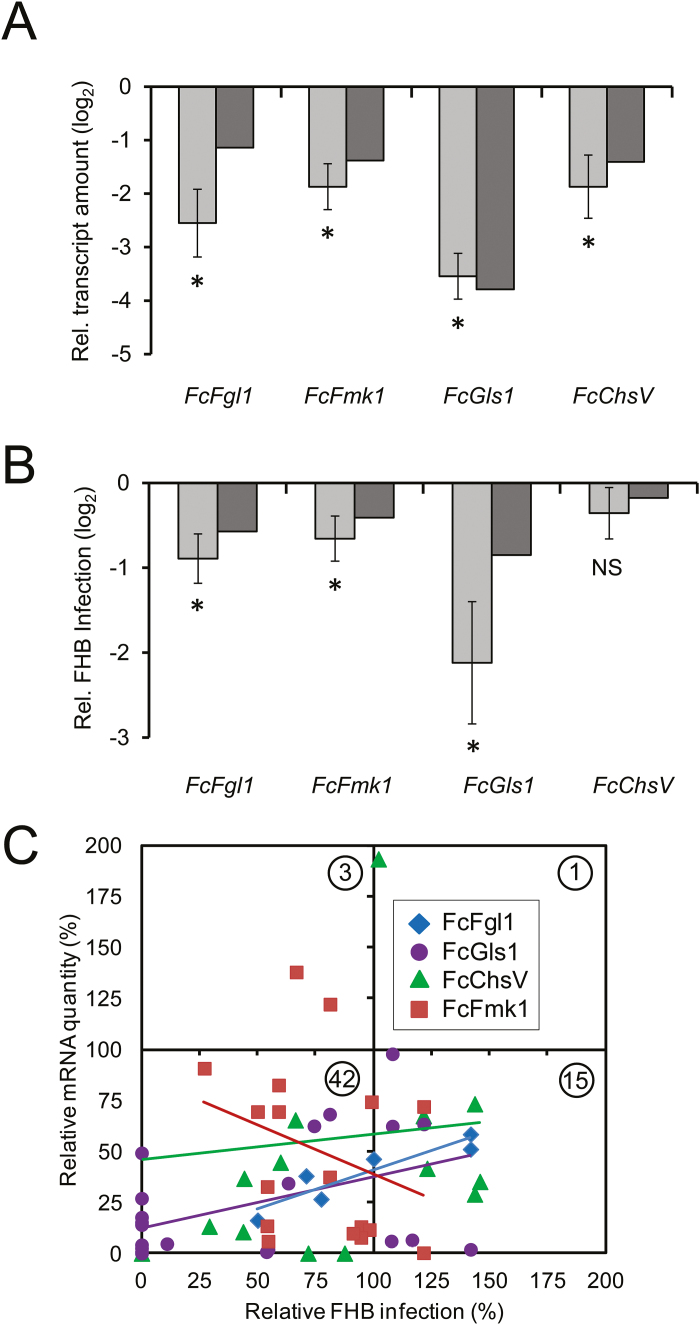
BSMV-mediated HIGS of fungal target genes causes partial protection of spikes from *F. culmorum* attack. (A) mRNA levels of four fungal target genes in BSMV-treated spikes were analyzed by quantitative PCR (RT-qPCR) [normalized to *F. culmorum translation elongation factor1* (*EF1*) and log(2) transformed] and showed significant knock-down compared with BSMV:00 controls (*P*-value<0.01, one-sample *t*-test, indicated by an asterisk). Light and dark gray columns correspond to mean and median values, respectively, from 2–4 independent inoculation experiments. (B) Reduced FHB infection relative to BSMV:00 controls by BSMV-mediated HIGS of fungal target genes [log(2)-transformed data]. Asterisks indicate significant reduction (*P*<0.01, Wilcoxon signed rank test). Light and dark gray columns correspond to mean and median values, respectively, from 3–5 independent inoculation experiments. (C) Linear regression of relative target gene mRNA levels and relative FHB infection upon BSMV-mediated HIGS. The numbers inside circles correspond to the number of paired data per sector. The correlation for plants pre-treated with BSMV:Fgl1_as and BSMV:Gls1_as was significant (*P*<0.05; *R*=0.87 and 0.38, respectively).

Based on information from fungal mutants, we selected four candidate genes of *F. culmorum* that are likely to result in reduced virulence upon silencing. First, *F. graminearum* mutants of the secreted lipase Fc*Fgl1* were described to have strongly reduced virulence on wheat (Voigt *et al.*, 2005). Secondly, knock-out mutation of the myosin motor domain-containing chitin synthase V (Fc*ChsV*) gene in the maize anthracnose fungus *Colletotrichum graminicola* strongly impaired vegetative growth as well as plant infection (Werner *et al.*, 2007). The third target gene corresponds to the Fc*Fmk1* gene whose product, a mitogen-activated protein (MAP) kinase, a member of the yeast and fungal extracellular signal-regulated kinase subfamily, is an essential factor for pathogenicity of *F. oxysporum* and *F. verticilloides* attacking tomato and maize, respectively (Di Pietro *et al.*, 2001; Zhang *et al.*, 2011). The essential function of the Fmk1 protein appears to be restricted to *in vivo* interactions because vegetative growth and *in vitro* conidiospore formation of *fmk1* null mutants of *F. oxysporum* were normal (Di Pietro *et al.*, 2001). Fourthly, mutation of the single-copy gene *glucan synthase 1* (*GLS1*) in *C. graminicola* was found to be lethal, whereas RNAi-mediated silencing in transgenic fungal strains caused severe developmental abnormalities and non-pathogenicity (Oliveira-Garcia and Deising, 2013). We point-inoculated wheat spikes, which were pre-infected with recombinant BSMV strains containing target gene sequences in antisense orientation, by *F. culmorum* macroconidia and observed significant reduction of the amounts of the corresponding target mRNA (Fig. 4A). The target mRNA reduction was associated with moderate reduction of spreading FHB symptoms in the case of Fc*Fgl1* and Fc*Fmk1*, and with stronger symptom reduction in the case of Fc*Gls1* (Fig. 4B). In contrast, no FHB disease reduction was found in the presence of the Fc*ChsV*-silencing vector, which might be explained by functional redundancy with other, not targeted chitin synthases in *F. culmorum* or by insufficient silencing of the Fc*ChsV* gene to achieve disease reduction. As shown in Fig. 4C, an association of target mRNA reduction and the reduction of FHB disease symptoms was found: for all four target genes, the observed distribution of data points significantly (χ^2^ *P*<0.0001) deviated from a random distribution that would be expected to be centered on the ‘0/0’ co-ordinates for both parameters. A large majority of spikes showed both reduced mRNA levels and reduced disease symptoms. Gene by gene analysis furthermore revealed a positive correlation between target mRNA levels and disease symptoms for Fc*Fgl1* (*P*=0.0005) and Fc*Gls1* (*P*=0.0318), whereas no significant correlation could be found for Fc*Fmk1* and Fc*ChsV*. This could reflect a threshold phenomenon with respect to the required extent of Fc*Fmk1* target mRNA reduction for preventing FHB infection, whereas, in the case of Fc*ChsV*, the negative result is in line with the absence of a significant HIGS-related phenotypic effect (Fig. 4B). We used the si-Fi software (http://www.snowformatics.com/si-fi.html) to search for putative off-targets of the VIGS constructs in 43 635 cDNA sequences of wheat used for the design of the Agilent 44K Gene Expression array (criteria: 21bp alignment, one mismatch allowed), with negative results. This further supports the conclusion that target mRNA reduction in the fungus and not any other indirect effect was responsible for the reduced infection. In summary, transient HIGS mediated by BSMV antisense strains significantly reduced target mRNA levels of all four analyzed genes and reduced FHB disease symptoms for three of them. We conclude that the BSMV-mediated transient HIGS system represents a useful and versatile pre-screening tool for potential HIGS targets in *F. culmorum*, prior to the more laborious and time-consuming generation of stable HIGS RNAi lines in wheat or barley.

### HIGS in *F. culmorum* mediated by transgenic wheat

Wheat immature embryos were transformed with binary HIGS vectors containing inverted repeats either of a single-target sequence against Fc*Gls1* or of a triple-target sequence against Fc*Gls1*, Fc*Fmk1*, and Fc*ChsV* (Supplementary Fig. S4). Based on disease rating data from preliminary greenhouse experiments using T_1_ plants of 18 transformation events, five events were selected to produce homozygous T_2_ lines for repeated experiments in the greenhouse (Supplementary Table S4). Because we obtained only four events carrying the single-target Fc*Gls1* construct and only one event showed reduced infection, a second event was chosen arbitrarily (WG224A/1E02) in order to retain the possibility of comparing two independent events per construct. Four out of the five selected lines also showed reduced infection by ~20–35% in the T_2_ generation, whereas one line (no. 17 from event WG226A/2E11) was as susceptible as the controls and therefore was used as an additional, negative control for the subsequent semi-open greenhouse experiments. The FHB infection pressure across all experiments for T_1_ and T_2_ generations was high, resulting in ~70% diseased spikelets of azygous controls by 21 d after inoculation (Supplementary Table S4).

**Fig. 5.**
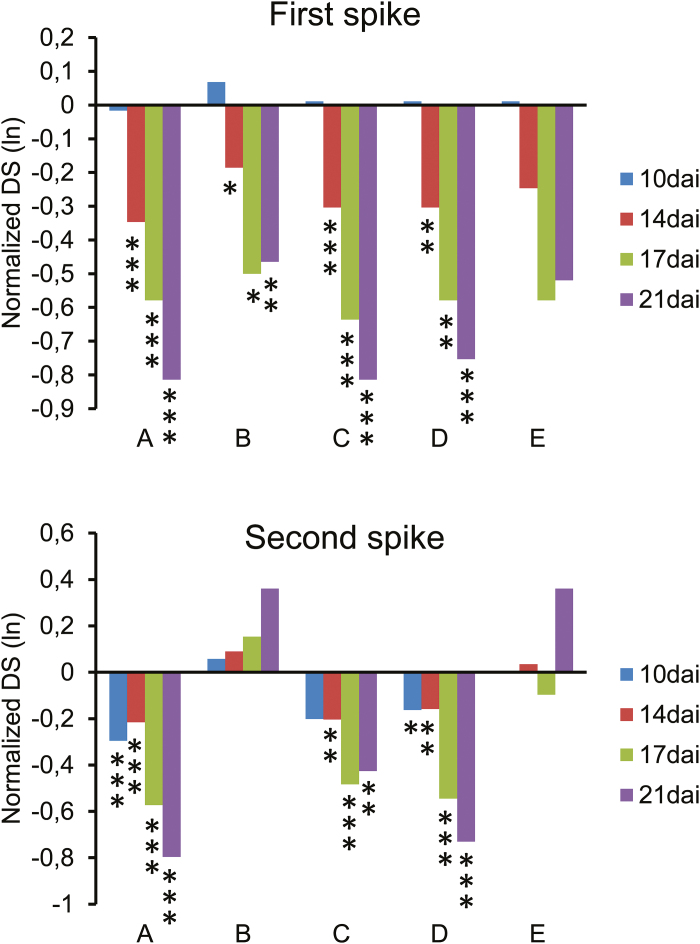
Quantitative protection from *F. culmorum* spike infection of transgenic wheat lines expressing HIGS constructs and growing under near-field conditions. Data of the semi-open greenhouse experiment show the median normalized disease severity [DS; log(e) transformed] for the first (main) and second spikes, as indicated. Four time points of DS were normalized to the corresponding mean of pooled azygous lines. A and B, and C–E refer to HIGS transgenic lines carrying the RNAi constructs pIPKb027_Gls1 and pIPKb027_CFG, respectively. Wilcoxon signed rank test was conducted, and a significant reduction of FHB infection of transgenic lines compared with azygous lines is indicated (*P<0.05; **P<0.005; ***P<0.0005).

The pre-selected transgenic lines were planted in semi-open greenhouses in order to be tested for resistance against *F. culmorum* under near-field conditions (Supplementary Fig. S5). Weather conditions during the FHB inoculation campaign in late spring 2013 were favorable for infection, with an average maximum daytime temperature of 23.6 °C during the first half followed by 27.3 °C during the second half of the experiment. Accordingly, we recorded high average infection rates of 36.2% and 72.0% diseased spikelets at 21 d after inoculation for the first and second developed spikes, respectively. Four out of the five lines selected for the semi-open greenhouse experiment showed significant protection of the first spikes from spreading FHB infection after log(e) transformation of relative disease severity normalized to a pool of azygous control plants (Table 2; Fig. 5). We also tested transgene effects by MANOVA of the log(e)-transformed absolute infection values, after having separated the data into the two spore preparations used for the earlier and later inoculations. Spore preparations turned out to be the most important factors for variability in terms of infection severity (Supplementary Table S2), but it remained unclear if these differences in infection severity were due to different virulence *per se* of the two spore preparations, or due to seasonal temperature increase. The analysis of absolute infection values revealed significant protection of at least one event per HIGS construct on the first and second spikes. Taken together, we observed a quantitative reduction of FHB disease symptoms in transgenic wheat lines carrying a HIGS construct against Fc*Gls1* or a triple-target construct against Fc*Gls1*, Fc*Fmk1*, and Fc*ChsV*. However, no obvious difference between the efficacies of the single-target versus the triple-target constructs was found.

**Table 2:** Details of transgenic lines selected for the semi-open greenhouse experiment

**Construct name short**	**Transgenic event**	**Generation**	**Line number**	**Group**	***n*** ^***a***^	**RI** ^***b***^ **(%)**	**Log(e) RI** ^***c***^
pIPKb027_Gls1	WG224A/1E02	T2	11	A	48	**44.3** ^***d***^	**–0.81** ^***d***^
	WG224B/2E06	T3	12	B	45	62.8	**–0.46** ^***d***^
pIPKb027_CFG	WG226A/2E13	T2	639	C	46	**44.3** ^***d***^	**–0.81** ^***d***^
	WG226A/2E13	T2	640				
	WG226A/2E10	T3	13	D	47	**47.1** ^***d***^	**–0.75** ^***d***^
	WG226A/2E11	T3	17	E	47	59.5	–0.52
Azygous lines	WG226A/2E08	T3		H–J	49	100	0.00
	WG226A/2E05	T2	Pool		45		
	WG184/1E08	T2			47		

^*a*^ Number of plants analyzed.

^*b*^ Median values of relative infection (% of azygous control plants corresponding to a pool of three lines).

^*c*^ Median values of log(e)-transformed relative infection (compared with azygous control plants represented by a pool of three lines).

^*d*^ Statistically significant disease reduction (Wilcoxon signed rank test, *P*<0.05) compared with pooled azygous control lines are highlighted in bold.

**Fig. 6.**
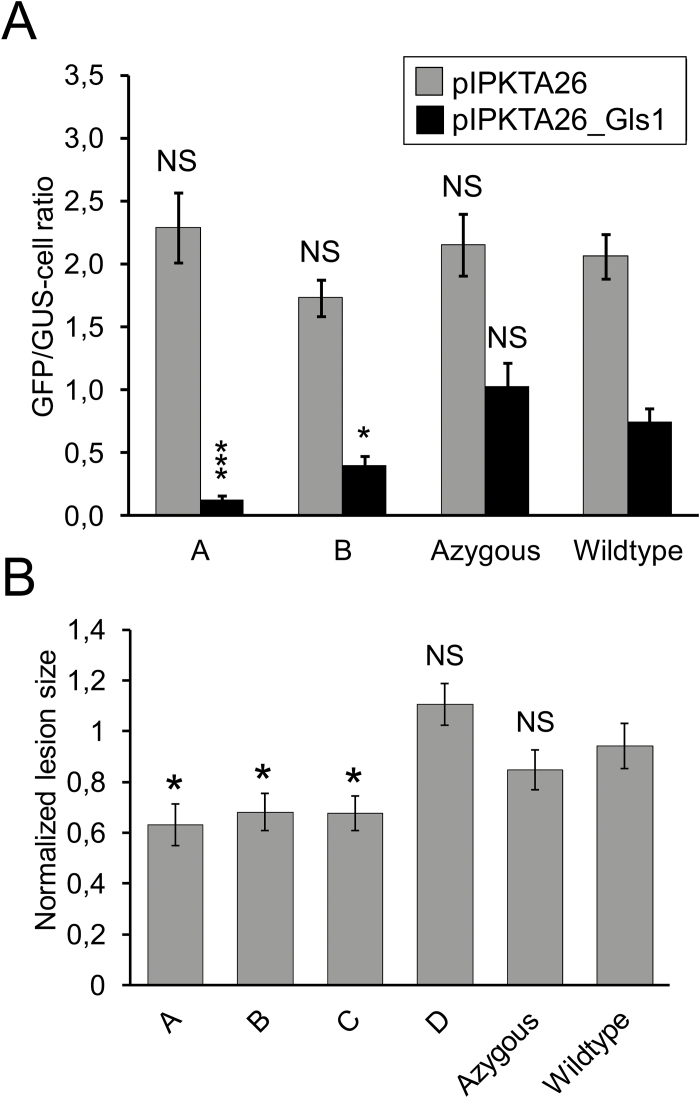
Quantitative protection from *F. culmorum* leaf infection and presence of RNAi activity in transgenic wheat leaves expressing HIGS constructs. (A) Detached second leaf segments of transgenic wheat lines were co-bombarded with a mixture of plasmids pIPKTA26_Gls1 and pUbiGUS for reporting the presence of silencing (small) RNAs and transformation efficiency, respectively. As a negative silencing control, the empty vector pIPKTA26 was co-bombarded together with pUbiGUS. The number of GFP-fluorescing cells was normalized to the number of GUS-stained cells per bombardment. The mean ±SEM of 14 transgenic plants derived from two independent bombardment series is shown. The statistical significance of differences from wild-type plants was calculated by two-tailed Student’s *t*-test. (B) Detached second leaf segments of transgenic wheat lines were needle-pricked and inoculated with *F. culmorum*. Four to five days after inoculation, disease was scored by quantifying the area of infected, necrotic lesions. The mean ±SEM from three independent inoculation experiments with an average of 55 inoculation sites per transgenic line. (A, B) Groups A, B, and C, D refer to HIGS transgenic lines carrying the RNAi constructs pIPKb027_Gls1 and pIPKb027_CFG, respectively. One and three asterisks correspond to significant difference from the wild-type control at *P*<0.05 and *P*<0.0005 levels, respectively; NS, not significant.

We propose that the observed protection in the four transgenic lines was due to HIGS of fungal target genes. To substantiate this hypothesis, we addressed transcript levels of Fv*Gls1*, which was targeted by both the single- and triple-HIGS constructs, in *F. culmorum* developing in transgenic plants. However, no consistent reduction or even transcript increase was observed, as reported before for several housekeeping HIGS target genes of *Puccinia striiformis* and *Blumeria graminis* (Nowara *et al.*, 2010; Yin *et al.*, 2011). This may reflect an inherent problem of selecting for those fungal cells that escaped (lethal) HIGS in the transgenic lines, which are likely to contain lower amounts of silencing RNA compared with the extremely efficient VIGS system. The latter assumption is based on our transcript quantification by quantitative real-time PCR in spikes of BSMV-infected wheat cv. Apogee, which contained 0.6- to 7.3-fold higher levels of BSMV α-fragment relative to the wheat GAPDH (glyceraldehyde phosphate dehydrogenase) internal reference, and comparing those with reported, ~10-to 100-fold lower, transgene mRNA levels in spikes of wheat cv. Bobwhite driven by the maize ubiquitin promoter, which was also used for the transgenic wheat plant reported here (Okubara *et al.*, 2002). To circumvent this problem, we developed a selection-neutral reporter assay for detecting RNAi activity in transgenic cells or entire plants. The RNAi reporter construct is based on an unstable version of GFP (Dong *et al.*, 2006) fused to an RNAi target sequence downstream from the GFP ORF. In the presence of silencing dsRNAs, the fusion mRNA will be cut, thereby preventing correct polyadenylation and leading to rapid transcript degradation (Supplementary Fig. S6). Bombardment of the reporter construct into cells transiently or stably expressing a corresponding RNAi construct would be predicted to cause a reduction of GFP expression, reflected by a reduced number of GFP-fluorescing cells. Proof of concept for the RNAi reporter assay was obtained by co-bombarding barley epidermal cells with pIPKTA30_Mlo for silencing of the barley *Mlo* gene together with pIPKTA26_Mlo for reporting RNAi effects. As shown in Supplementary Fig. S7, the number of GFP-fluorescing cells was significantly reduced in the presence of an RNAi construct targeting *Mlo*. The silencing of *Mlo* has been shown to phenocopy *mlo* mutations in barley with strong resistance against the powdery mildew fungus *B. graminis.* As expected, the susceptibility index (SI) of pIPKTA30_Mlo-bombarded cells was also significantly reduced (Douchkov *et al.*, 2005). Interestingly, the effect on both GFP-fluorescing cell number and the SI gradually diminished in parallel by using RNAi constructs with increasing numbers of random mutations of *Mlo*, thereby simulating reduced RNAi efficiency by evolutionary target distance. Using the validated RNAi reporter system, we tested transgenic wheat lines for RNAi activity against Fc*Gls1* in detached leaf segments (Fig. 6A). In the case of the single-target Fc*Gls1* construct, a significant reduction in GFP numbers was observed. No reproducible reduction was obtained in plants carrying the triple-RNAi construct. This is consistent with our previous finding of limited efficiency of RNAi in transient assays resulting in a strong drop of efficiency by combining more than one target per cell (Douchkov *et al.*, 2005). We also tested if *F. culmorum* infection was reduced in detached leaves after wound inoculation, and found significant reduction of lesion size in three out of the four selected lines that gave rise to reduced infection in spikes (Fig. 6B). In summary, both selected single-target transgenic lines possessed RNAi activity against Fc*Gls1* and caused reduced infection in detached seedling leaves.

**Fig. 7.**
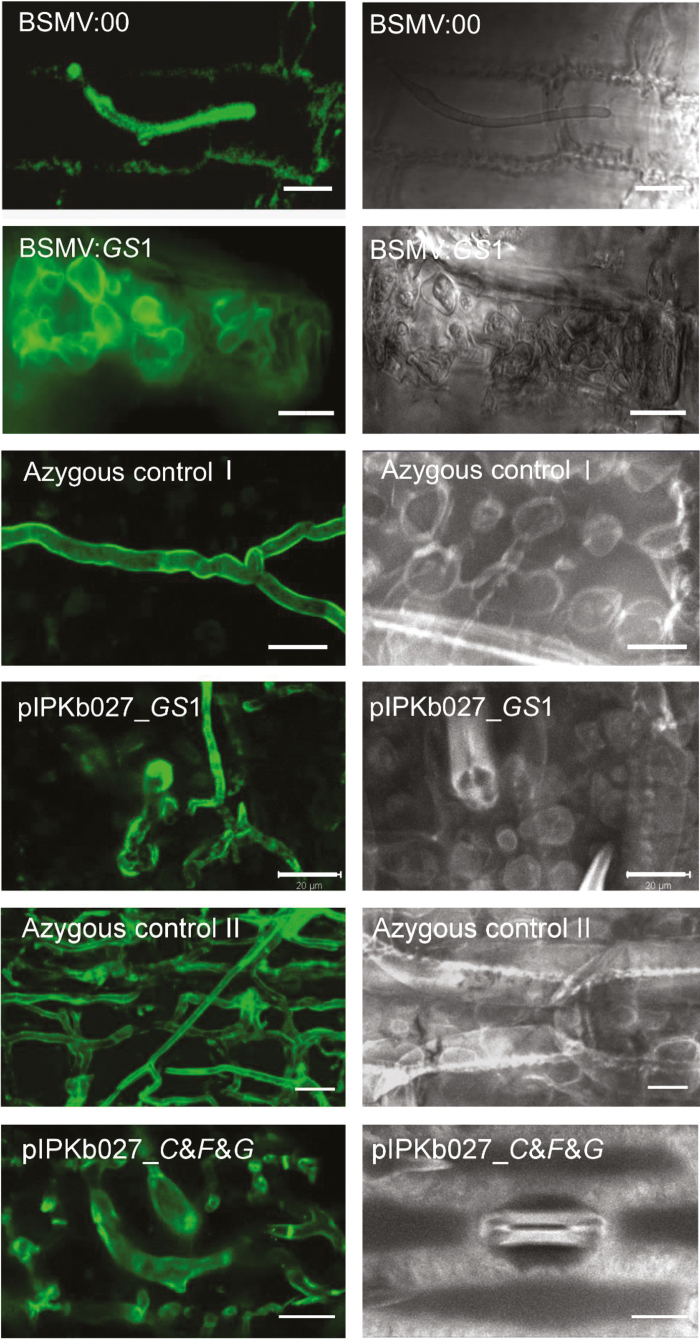
HIGS of glucan synthase 1 in *F. culmorum* leads to target-specific cell wall aberrations. Left-hand panels: wheat germ agglutinin (WGA) staining of *F.culmorum*-infected palea tissue analyzed by confocal microscopy. BSMV:00 and BSMV:Gls1, palea samples of spikes pre-infected with the corresponding BSMV strains and stained 3 d after *F. culmorum* inoculation (scale bar=10 µm). pIPKb027_Gls1 and pIPKb027_CFG, palea samples of HIGS transgenic spikes stained 9–14 d after *F. culmorum* inoculation (scale bar=20 µm). Azygous control 1 and 2 show normal hyphal development of *F. culmorum* in azygous control lines grown together with pIPKb027_Gls1 and pIPKb027_CFG, respectively. Right-hand panels show corresponding bright-field images.

Stable RNAi events of *F. solani* and *C. graminicola* targeting *Gls1* resulted in lysis of spores and hyphae, abnormal hyphal morphology such as strongly reduced cell elongation rates, and a bulbous increase in hyphal diameter (Oliveira-Garcia and Deising, 2013). This phenotype is probably caused by the reduction of β-glucan biosynthesis, which is known to be essential for resistance of the hyphal cell walls against internal turgor pressure. We examined hyphae of *F. culmorum* growing in spikes of BSMV:Gls1_as-infected plants or in transgenic HIGS lines targeting Fc*Gls1* for a phenocopy of this abnormal growth behavior. As shown in Fig. 7, moderate to severe distortions of *F. culmorum* hypal morphology resembling those observed in stably silenced *C. graminicola* were found in spikes of wheat transiently or stably expressing Fc*Gls1*-targeting HIGS constructs. This suggests that the observed disease reduction by *FcGls1*-silencing constructs was due to true target mRNA degradation rather than off-target effects.

## Discussion

Protection of wheat against *F. culmorum* by HIGS was tested in transient gene silencing experiments and in transgenic lines after having established appropriate assay systems and plant growth conditions. BSMV-mediated transient HIGS of target genes in the powdery mildew fungus *B. graminis*, in *F. graminearum* attacking *A. thaliana* or barley, and in the stripe-rust fungus *P. striiformis* was recently reported (Nowara *et al.*, 2010; Koch *et al.*, 2013; Panwar *et al.*, 2013) and should be extended here to the wheat–FHB interaction. Because spikes or cobs of wheat, barley, and maize are the agronomically most important plant parts infected by FHB fungi causing yield reduction as well as toxin contamination of kernels, we established a BSMV-mediated HIGS system in adult plants. The throughput of this transient assay system was increased by using the early flowering extreme dwarf wheat cv. Apogee (Fig. 1A), which was described as highly susceptible to FHB, therefore being well suited for stringent validation of potentially resistance-enhancing QTLs, defense-related genes, or gene silencing constructs (Mackintosh *et al.*, 2006). The rationale behind the experiment in the semi-open greenhouses was to test stable HIGS of *F. culmorum* target genes pre-selected by VIGS for its efficiency under near-field conditions provided by these facilities. First, seedlings were planted into natural soil brought from a nearby field site, without limitation of horizontal or vertical root development. Secondly, the greenhouses containing metal mesh on two opposite sides allow free air convection, which results in similar daily temperature profiles inside and outside the greenhouses.

Wheat plants transiently or stably expressing HIGS transgenes that target essential genes for pathogenicity or growth of the FHB fungus *F. culmorum* were significantly protected from the disease. Thus, the biotechnological approach of using potentially harmful sequence information instead of, for example, antifungal chemistry and expressing it in transgenic plants may be applicable to protect wheat from one of its most damaging fungal pathogens. Our results are in line with and extend beyond recent reports about protection of barley and wheat stably expressing HIGS constructs against the other major FHB-causing fungus *F. graminearum* (Koch *et al.*, 2013; Cheng *et al.*, 2015). The HIGS targets chosen for these studies encode the cell wall biosynthetic enzyme chitin synthase III and the major fungicide target P450 lanosterol demethylase catalyzing a key step in ergosterol biosynthesis, respectively. Taken together, all studies indicate that HIGS is not restricted to controlling biotrophic pathogens, for which positive results have accumulated over the last few years (Nowara *et al.*, 2010; Zhang *et al.*, 2012; Panwar *et al.*, 2013; Pliego *et al.*, 2013), but may also become important to control hemibiotrophic or even necrotrophic pathogens.

The average degree of protection observed with the different HIGS constructs was not complete, although a certain percentage of single spikes was always fully protected from disease spread, showing no visible symptoms as late as 21 d after inoculation. This indicates that HIGS is a quantitative rather than a threshold phenomenon, in line with the manifold reported quantitative nature of RNAi as the likely underlying mechanism. Likewise for all analyzed HIGS targets, we observed a range of weak to complete reduction of target transcript levels in individual spikes of BSMV-infected plants. On average, significantly protected transgenic lines in the semi-open greenhouse trial showed 50–60% fewer FHB symptoms when directly compared with the median values of azygous control lines (Fig. 5). This level of disease reduction is considerable and corresponds approximately to the combined effect of two major QTLs for FHB resistance derived from Chinese landrace Sumai 3 after introgression into susceptible wheat genotypes (von der Ohe *et al.*, 2010; Salameh *et al.*, 2011). It should be noted that the recipient wheat cultivars Apogee and Bobwhite used for BSMV-mediated HIGS and stable transformation with HIGS constructs, respectively, are highly susceptible to the disease, rendering its control more difficult. It would therefore be interesting to test if backcrossing of selected lines obtained in cv. Bobwhite into contemporary, moderately FHB-resistant wheat cultivars could improve HIGS transgene efficacy, aiming at a level of protection which might be attractive for the development of transgenic cultivars (Mumm, 2013). Irrespective of background optimization for a stable transgenic HIGS approach, the moderate level of protection reported here poses the question of possibilities for further improving its efficiency. First of all, a careful selection of fungal target genes is probably the most essential prerequisite for success. We based our choice of HIGS targets on reported results of mutants in fungal species other than *Fusarium* ssp. with severely reduced virulence (*Fgl1*, *Fmk1*, and *ChsV*) or on non-pathogenic stable RNAi strains (*Gls1*). Although such selection criteria appear reasonable, they might suffer from limited comparability of gene function across pathogen species, or from species-specific functional redundancy of non-targeted gene paralogs. Other factors possibly affecting HIGS efficiency are the abundance and turnover rate of target mRNA or its accessibility to HIGS-related siRNAs depending on three-dimensional RNA structure. By using transgenic *F. graminearum* strains expressing a series of HIGS constructs directed against different regions of *ChsIII*, Cheng *et al.* (2015) identified the most efficient silencing construct that was then successfully used for the generation of stable transgenic wheat with enhanced FHB resistance. Because the generation of large numbers of stable transgenic wheat lines is still a laborious and time-consuming process, transient silencing in BSMV-pre-infected, flowering wheat plants represents a versatile tool to pre-screen and identify efficient targets. In theory, the targeting of multiple genes via chimeric hairpin constructs might result in stronger protection phenotypes, compared with a single-target approach. However, our results from triple-target HIGS lines of wheat showed no substantial gain in protection compared with the single-target construct. This might be due to limited availability of RNA-degrading protein (complexes) in the host or the fungus, which—when stochastically distributed over three populations of siRNA molecules—resulted in poor degradation of the individual target mRNAs. Similar results of missing additive or synergistic effects of multitarget RNAi constructs were also obtained for a number of barley and *B. graminis* (powdery mildew) genes (DN and PS, unpublished results). Undersampling due to limited numbers of independent transgenic events generated per construct might be another, more trivial reason why we had not identified more stongly protected HIGS lines.

The nature of the HIGS-mediating RNA molecules (antisense RNA, long dsRNA, or siRNA) as well as the route by which the active HIGS principle is delivered to FHB fungi or other pathogens are still unknown. In animals and human, exosomes derived from hepatic, mast, or B cells were found to shuttle mRNA and small RNAs into recipient cells (Valadi *et al.*, 2007; Pan *et al.*, 2012; Wahlgren *et al.*, 2012). In plants, exosomes were found at pathogen encounter sites, but it is not known if they also contain (small) RNAs. Nevertheless, these observations open up the question of the existence of an evolutionarily conserved mechanism of RNA-based cell–cell communication that might have evolved first as a defense strategy against pathogens. Irrespective of these open questions, recent data suggest that the exchange of silencing molecules across interacting organisms may be a naturally occurring phenomenon. First, transposon-derived, small RNA-based effectors targeting defense-related plant transcripts and promoting disease have been identified in the fungus *Botrytis cinerea* (Weiberg *et al.*, 2013). Secondly, the oomycete pathogen *Phytophthora sojae* was described to express host RNAi suppressors in *Nicotiana bentaminiana* and soybean similar to viral pathogens (Qiao *et al.*, 2013). Such suppressors might act on miRNA-mediated host defense responses or on their (speculative) HIGS mechanism. In the light of these recent findings, a systematic sequence search for non-coding RNA molecules of host plant species matching regulatory or protein-encoding RNAs of interaction organisms such as fungal pathogens might result in the discovery of natural, HIGS-triggering RNAs, with the option to select for favorable host alleles promoting HIGS efficacy in (pre-)breeding approaches.

## Supplementary data

Supplementary data are avaliaible at *JXB* online

Figure S1. Plasmid map of RNAi reporter construct pIPKTA26_SwaI.

Figure S2. Schematic diagram of BSMV-HIGS constructs used.

Figure S3. *ARF2-like* RNAi target prediction by si-Fi software.

Figure S4. Binary HIGS constructs for transgenic wheat.

Figure S5. Details of the semi-open greenhouse facility for the near-field trial.

Figure S6. Flow chart of the GFP-based reporter assay for RNAi of barley *Mlo*.

Figure S7. Proof of concept for the RNAi reporter assay by silencing the barley *Mlo* gene.

Figure S8. Original images of the upper and lower part of the same agarose gel used for separation of PCR fragments shown in Fig. 3A.

Table S1. PCR primers and TaqMan probes used.

Table S2. Detailed data from statistical analysis of absolute and log(e)-transformed normalized infection values from the trial in semi-open greenhouses under near-field conditions.

Table S3. Primary data (signal intensity values) of transcript levels derived from the 44K Wheat Gene Expression microarray of Agilent Co.

Table S4. Disease scoring of transgenic events of the T_1_ and T_2_ generation in a standard greenhouse.

Supplementary Data
