# Supplementary material for: Host-induced silencing of Fusarium culmorum genes protects wheat from infection
Source: J Exp Bot. 2016 Aug 18;67(17):4979–91. doi: 10.1093/jxb/erw263 (PMC5014151; doi:10.1093/jxb/erw263)
Supplement: Supplementary Data [file supp_67_17_4979__index.html]

Host-induced silencing of Fusarium culmorum genes protects wheat from infection — Host-induced silencing of Fusarium culmorum genes protects wheat from infection — Supplementary Data 

# Host-induced silencing of *Fusarium culmorum* genes protects wheat from infection

## Supplementary Data

Data files

- supplementary\_figures\_S1\_S8.pdf - Supplementary Data
- Supplementary\_Table\_S1.xlsx - Supplementary Data
- Supplementary\_Table\_S2.xlsx - Supplementary Data
- Supplementary\_Table\_S3.xlsx - Supplementary Data
- Supplementary\_Table\_S4.xlsx - Supplementary Data
