# Supplementary material for: Host-induced silencing of Fusarium culmorum genes protects wheat from infection
Source: J Exp Bot. 2016 Aug 18;67(17):4979–91. doi: 10.1093/jxb/erw263 (PMC5014151; doi:10.1093/jxb/erw263)
Supplement: Supplementary Data [file supp_erw263_supplementary_figures_S1_S8.pdf]

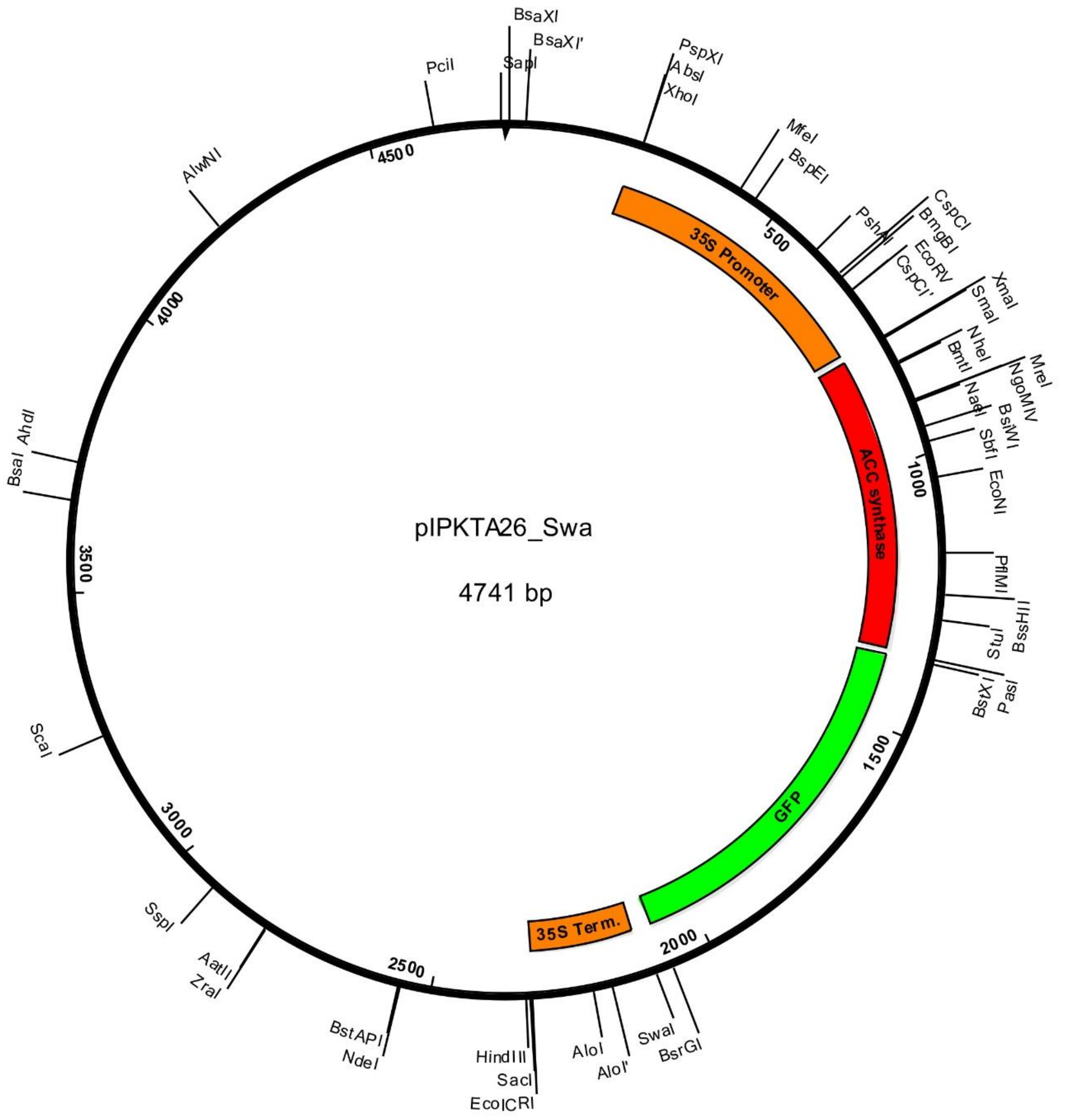

Figure S1: Plasmid map of RNAi-reporter construct pIPKTA26_SwaI.

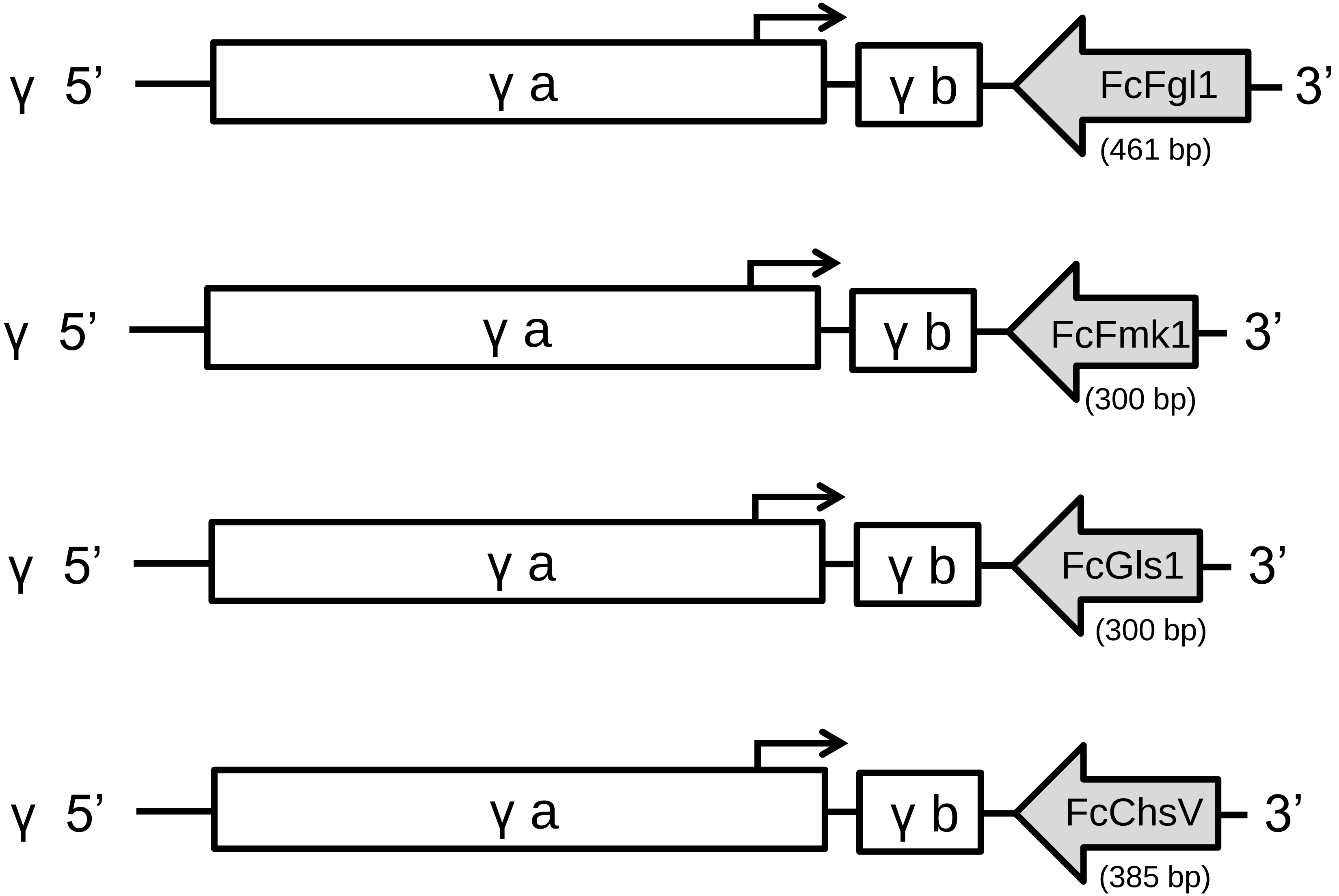

Figure S2: Schematic representation of recombinant γ RNA of BSMV expressing the four selected HIGS target mRNAs in antisense orientation.

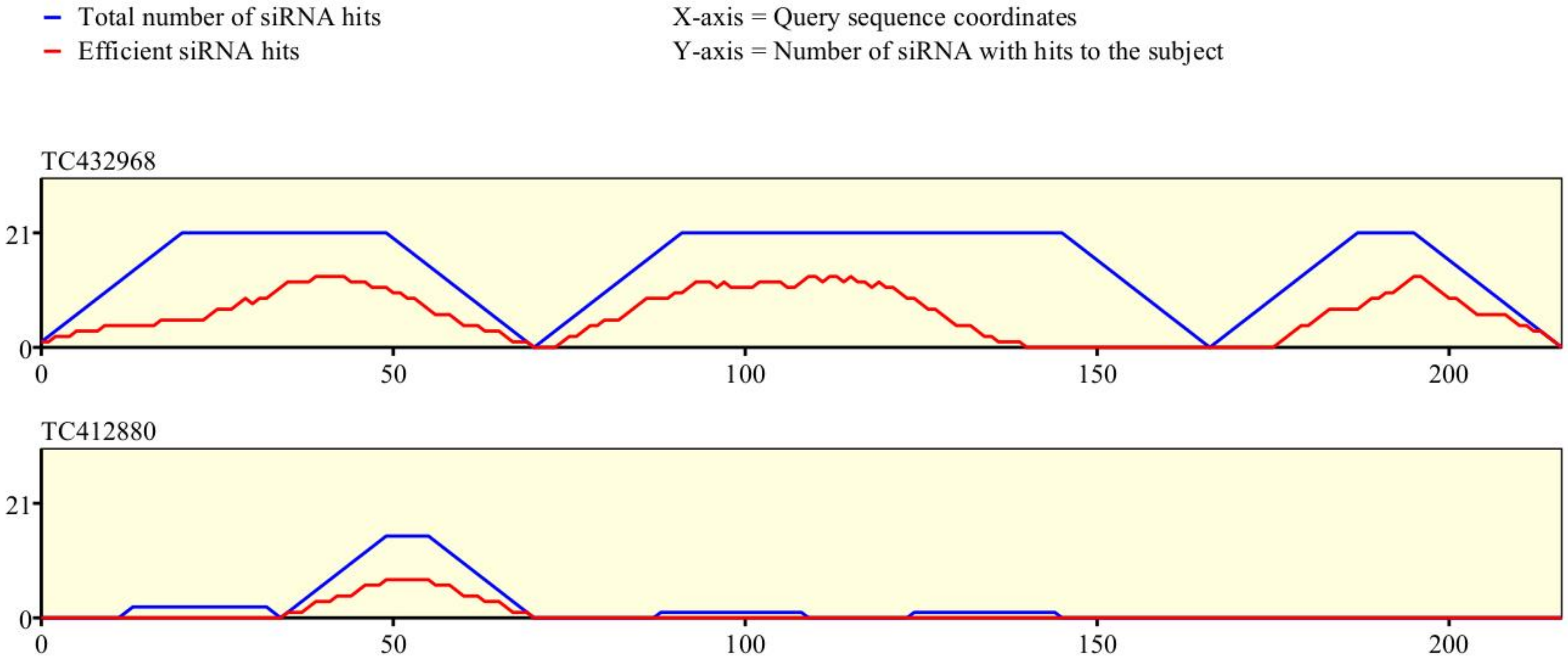

Figure S3: *In silico* prediction of VIGS target sequences for BSMV:Arf2_as.
Target predition was done by SiFi software. TC432968 corresponds to the closest wheat homolog of the AtARF2 protein and is represented by probe A_99_P502902 of the Agilent 44K Wheat Gene Expression microarray. TC412880 corresponds to a wheat *Arf2-like* sequence (Acc. AY902381) annoted as TaArf2 in GenBank and is represented by Agilent probes A_99_P172264 and A_99_P211316. Two additional VIGS-targeted Arf2-like cDNA sequences of the wheat gene index without Agilent probe representation on the 44K array are not shown.

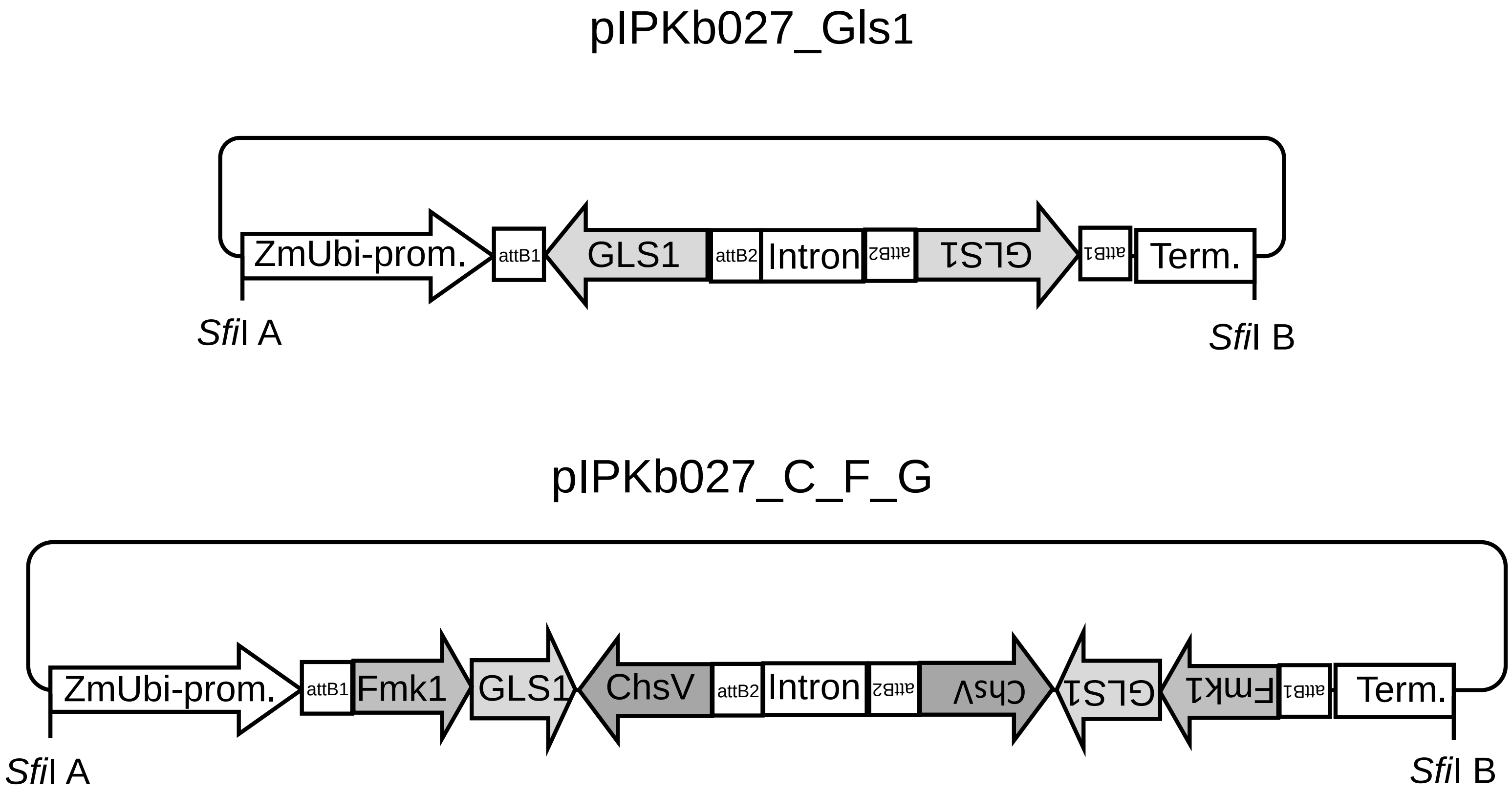

Figure S4: Schematic representation of the silencing cassettes in binary vector pIPKb027 (Acc. KJ508866) used for HIGS in stably transformed wheat plants.
ZmUbi-prom., N.N-bp promoter of the maize *Polyubiquitin1* gene (Christensen et al., 1992); Intron, wheat RGA2 intron (Acc. AF458273); Term., terminator of the pea Rubisco small subunit E9 gene (Acc. J01257).

Figure S5: Details of the semi-open greenhouse experiment conducted in 2013.

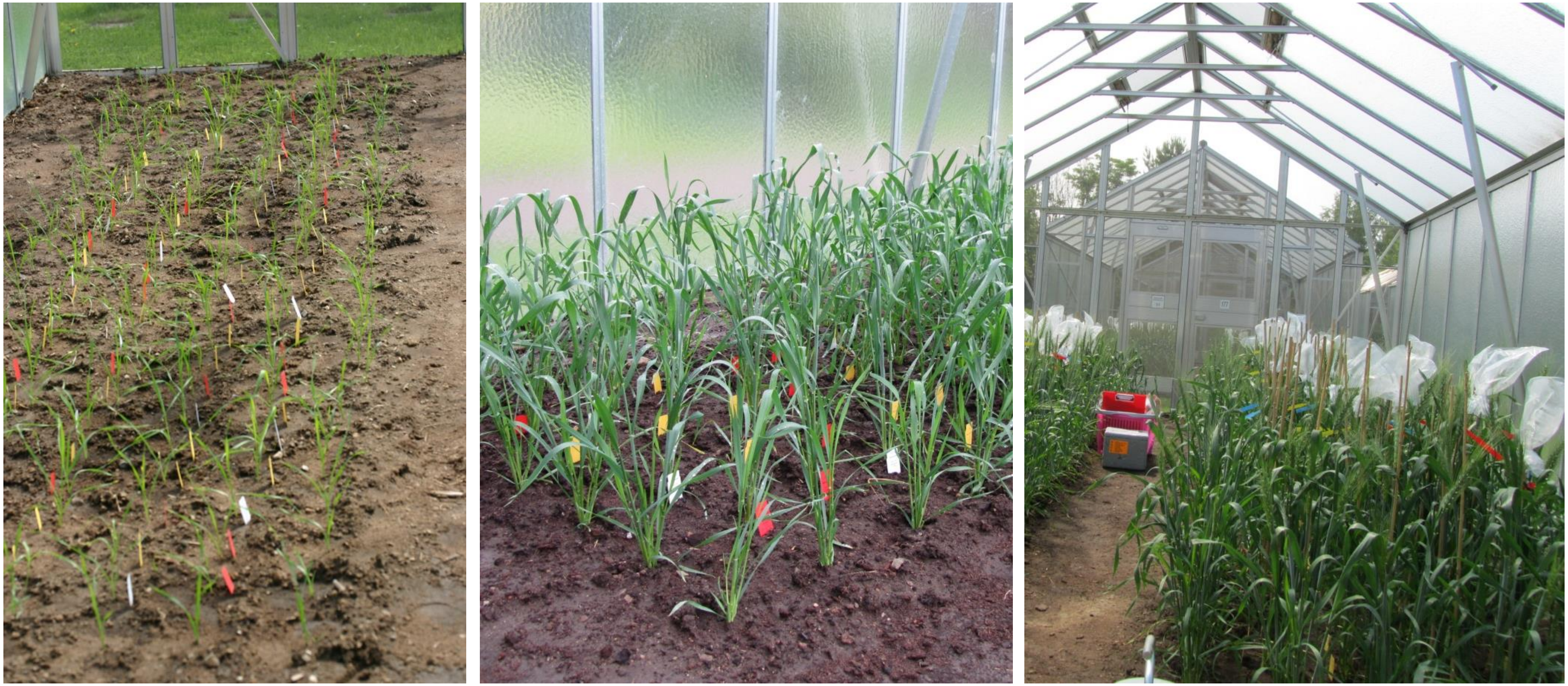

4 Weeks after sowing (1 week in semi-open-greenhouse)

8 Weeks after sowing (vegetative plant development)

10 Weeks after sowing (start of FHB inoculation)

Figure S5 (cont): Details of the semi-open greenhouse experiment conducted in 2013.

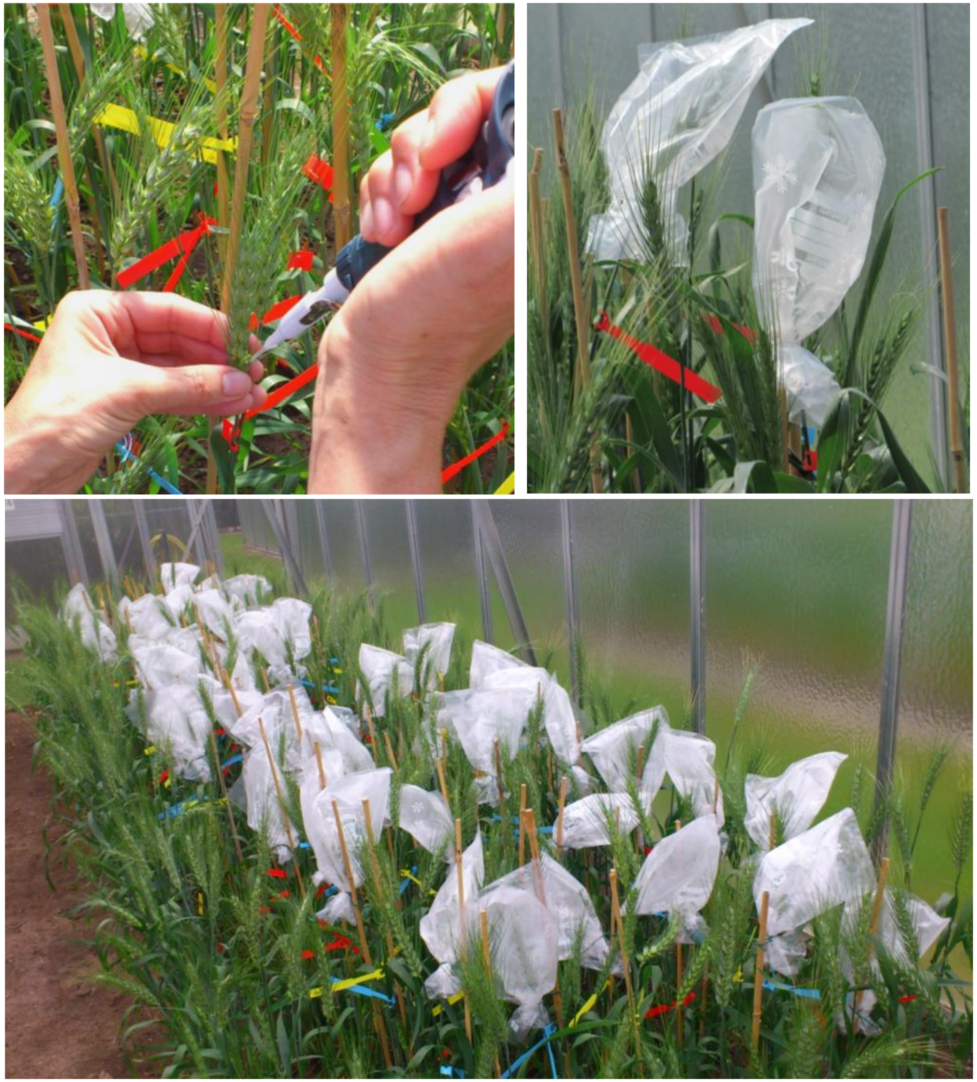

FHB point inoculation into floret (1000 conidia/ floret).

Inoculated spikes were covered by transparent plastic bags for 48 h.

The inoculation dates were marked by differently coloured labels.

Figure S5 (cont): Details of the semi-open greenhouse experiment conducted in 2013.

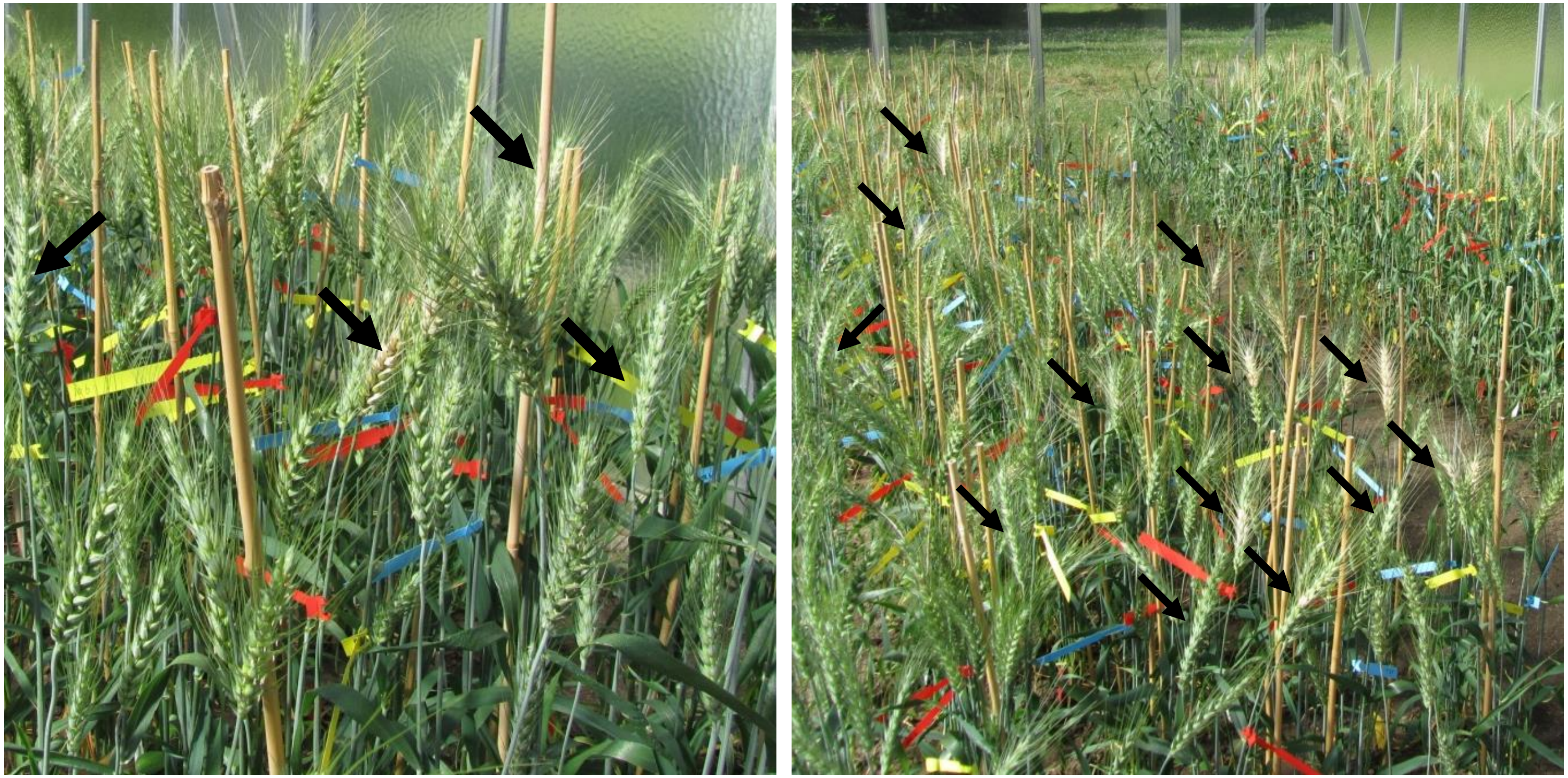

FHB disease symptoms at different times after inoculation, depending on flowering time and inoculation date per plant (10~21 d after inoculation). Arrows point at severly blighted spikes.

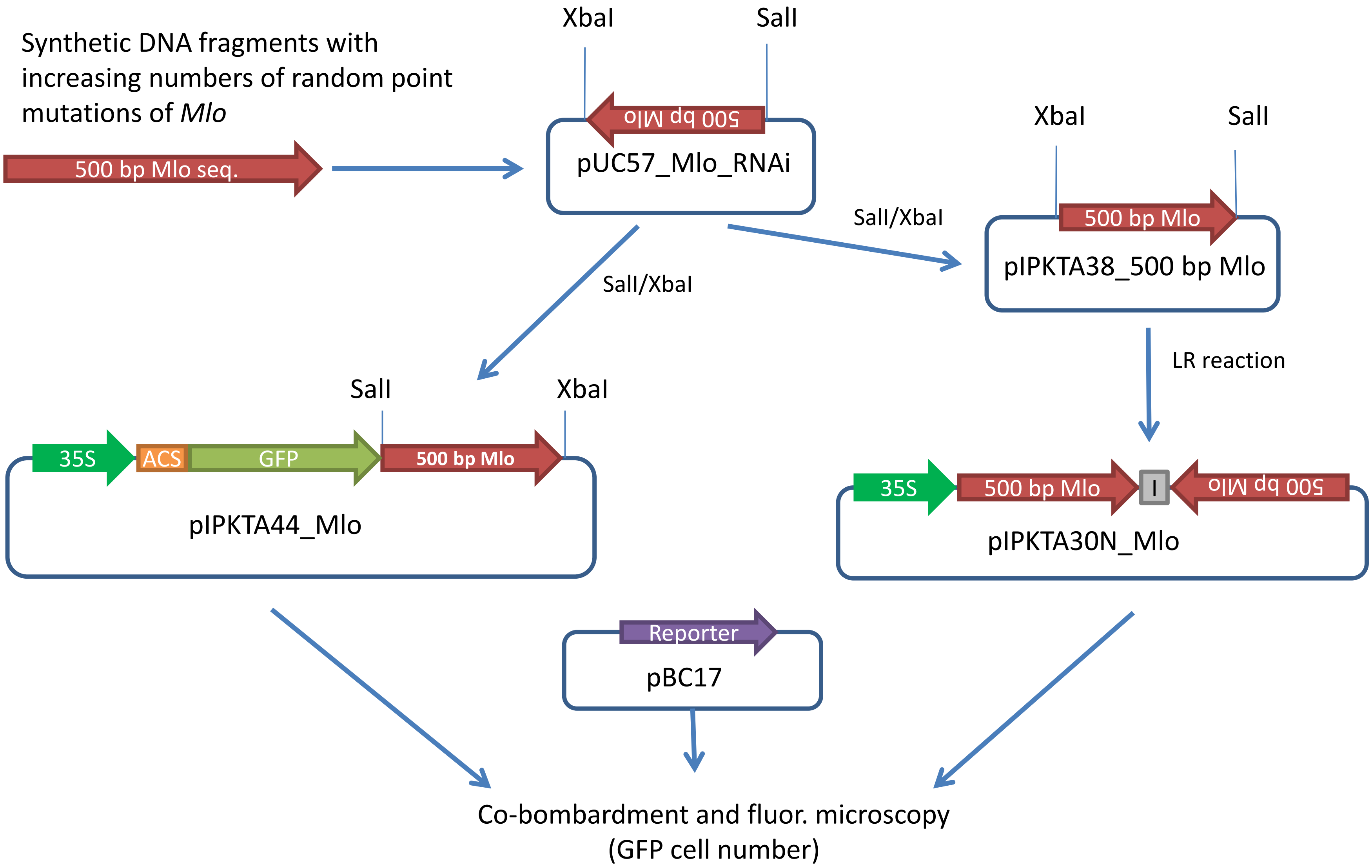

Figure S6: Flow chart of plasmid constructions and co-bombardment for GFP-based reporting of *Mlo*-RNAi activity in bombarded barley epidermal cells.
ACS, ACC synthase N-terminal sequence; 35S, CaMV 35S promoter of approximately 500 bp length; The CaMV 35S terminator in pIPKTA30N and pIPKTA44 is not shown.

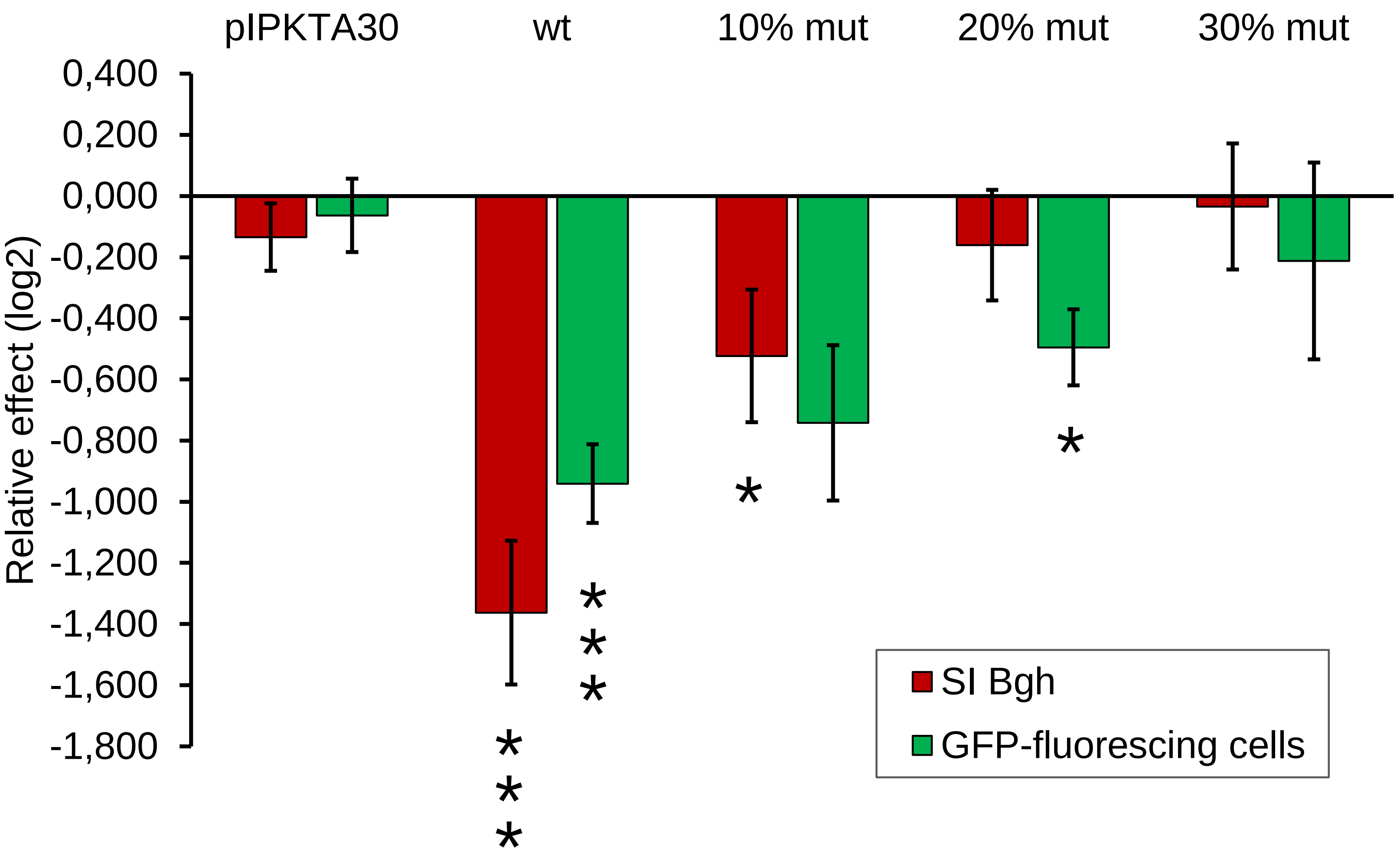

Figure S7: Proof of concept for the RNAi-reporter cassette based on an instable GFP version fused transcriptionally to a corresponding RNAi-target sequence.

A fragment of the barley *Mlo* gene was cloned into the reporter cassette resulting in pIPKTA44_Mlo and co-bombarded with *Mlo* RNAi constructs in pIPKTA30 containing 500 bp of wildtype *Mlo* mRNA sequence or versions with increasing numbers of random point mutations. As a third component, pBC17 leading to anthocyan accumulation was bombarded for the normalization of transformation efficiency (Schweizer et al., 2000). Fold reduction of the anthocyan-normalized number of GFP-fluorescing cells and of the susceptibility index (SI) against the powdery mildew fungus *B. graminis* caused by *Mlo* silencing is shown.

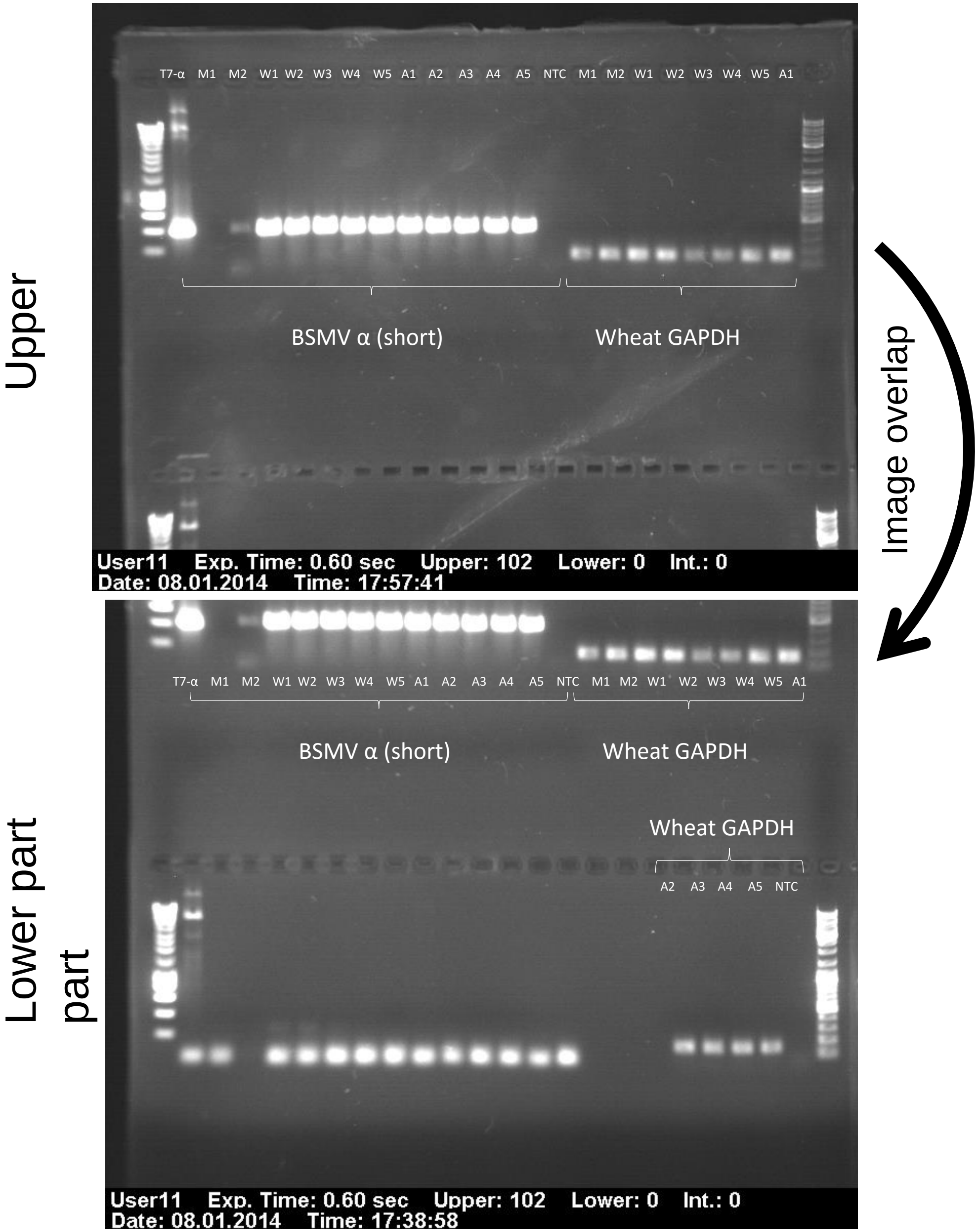

Figure S8: Original images of upper and lower part of the same agarose gel used for separation of PCR fragments shown in Figure 3A.
DNA size standards: Leftmost lane, SmartLadder" (Eurogentec Co.); rightmost lane, "GeneRuler DNA Ladder Mix" (ThermoFisher Scientific Co.).
